# Exploring Alternative Measurements of Cardiorespiratory Fitness in Patients With Mild Ischemic Stroke at Acute Phase

**DOI:** 10.3389/fneur.2022.801696

**Published:** 2022-02-09

**Authors:** Qingming Qu, Jie Zhu, Hewei Wang, Qi Zhang, Yongli Zhang, Zhijie Yan, Qiwei Fan, Yuanyuan Wang, Ying He, Liqing Yao, Lijuan Xu, Chao Zhang, Jie Jia

**Affiliations:** ^1^Department of Rehabilitation Medicine, Fudan University Huashan Hospital, Shanghai, China; ^2^National Center for Neurological Disorders, Shanghai, China; ^3^Department of Rehabilitation Medicine, The People's Hospital of Jiaozuo City, Jiaozuo, China; ^4^Department of Rehabilitation Medicine, The Second Affiliated Hospital of Kunming Medical University, Kunming, China; ^5^Linping Hospital of Integrated Traditional Chinese and Western Medicine, Linping, China; ^6^Hangzhou Xiaoshan Neighborhood United Hospital, Hangzhou, China; ^7^National Clinical Research Center for Aging and Medicine, Fudan University Huashan Hospital, Shanghai, China

**Keywords:** cardiorespiratory fitness, grip strength, six-minute walk test, cardiopulmonary exercise test, mild ischemic stroke

## Abstract

**Background:**

While emerging studies have suggested an association of cardiorespiratory fitness (CRF) with stroke risk and overall health outcomes, little is known regarding the optimum methods of CRF measurement in patients with mild acute ischemic stroke.

**Objective:**

The aim of this study was to explore the association between the 6-min walk distance (6MWD) and other measurements related to CRF in patients with mild ischemic stroke at the acute stage.

**Methods:**

A total of 30 patients with stroke and 71 healthy subjects matched for age and grip strength (GS) were prospectively recruited. All patients were within 14 days after stroke onset and presented mild motor impairment (with a full score of Fugl-Meyer Motor Assessment). Demographic data of both groups and clinical information of the stroke group were documented, and the CRF comparison between the two groups was conducted. Each participant underwent a one-time assessment of 6MWD and a series of measurements related to CRF, including GS, 10-m walk test (10mWT), five-times sit-to-stand time (FTSST), functional reaching test (FRT), Berg Balance Scale (BBS), and waistline. Pearson's product-moment correlation coefficient test and multiple linear regression were performed to explore the indicators of CRF.

**Results:**

Significant moderate correlations (0.3 < *r* <0.6) were found between 6MWD and GS of left hand (GS-left) (*r* = 0.573, *p* = 0.001), GS of right hand (GS-right) (*r* = 0.524, *p* = 0.003), FTSST (*r* = −0.551, *p* = 0.002), 10mWT (*r* = 0.554, *p* = 0.001), and FRT (*r* = 0.449, *p* = 0.021) in the patient group. While 6MWD displayed significant moderate correlations with waistline (*r* = 0.364, *p* = 0.002), 10mWT (*r* = 0.512, *p* < 0.001), FTSST (*r* = −0.573, *p* < 0.001), and FRT (*r* = 0.550, *p* < 0.001) in the healthy group. All these dependent variables were entered into a stepwise multiple linear regression analysis to evaluate their values in estimating CRF as measured by 6MWD in each group. Analyses suggested that GS-left (*p* = 0.002) and FTSST (*p* = 0.003) were the indicators of CRF in the patient group with stroke and explained 51.4% of the variance of 6MWD (*R*^2^ = 0.514); FTSST (*p* < 0.001), 10mWT (*p* < 0.001), and FRT (*p* = 0.021) were the indicators of CRF in the healthy group and explained 58.9% of variance of 6MWD (*R*^2^ = 0.589).

**Conclusions:**

Our data confirmed that CRF is impaired in patients with mild ischemic stroke at the acute phase. Moreover, GS-left may be an optional indicator of CRF in patients with mild acute ischemic stroke, but not in healthy people.

**Clinical Trial Registration:**

www.chictr.org.cn, identifier: ChiCTR2000031379.

## Introduction

Despite the rapid development of modern medicine, stroke remains a leading cause of adult mortality and disability in China and worldwide (1). Cardiorespiratory fitness (CRF), represented by peak oxygen uptake (VO_2peak_), is closely related to an individual's health status. Low CRF level is an independent risk factor for all-cause mortality (2), and a reliable predictor of poor functional prognosis and mortality after stroke (3). Emerging studies suggest that the improvement of CRF may provide benefits for walking capacity (4) and long-term recovery in patients with subacute and chronic stroke (5). However, for various factors, such as physical inactivity (6) (updated) before and after stroke (7), effects of the stroke itself, and the hospital environment (8), the VO_2peak_ level of stroke survivors decreases by about 47% compared with age- and sex-matched healthy subjects (9). Insufficient CRF causes an obvious difficulty in motor function rehabilitation early after stroke because motor relearning and neural network reorganization rely on repetitive training (10), and patients with decreased CRF levels may be less able to tolerate the highly intensive task training due to fatigue and rapid exhaustion (11).

Since CRF has a great impact on stroke outcomes, it is imperative to develop efficient methods of CRF measurement. The VO_2peak_, which is measured quantitatively by performing the cardiopulmonary exercise test (CPET), is widely accepted as the gold indicator of CRF (12). In practice, however, CPET is very costly, requiring expensive equipment, a specialized environment, and well-trained accessors, which makes it not applicable in a lot of small- and medium-sized medical institutions (13). Moreover, the spasticity, muscle weakness, coordination problem, and other limitations may preclude patients with stroke with moderate to severe impairment from safely and effectively undergoing CPET assessment (14). As an alternative, the American Stroke Association Scientific Statement recommends the use of a walking test for the assessment of CRF in stroke survivors (15). One of the most widely used practical simple walking tests in clinical use is the 6-min walk test (6MWT) (16), which demonstrates good to excellent test–retest reliability, good validation, and good responsiveness in patients with stroke (17, 18). In comparison with CPET, the 6MWT does not require a set of complex devices as well as the technical expertise of assessors (19). More importantly, previous studies have shown that the 6-min walk distance (6MWD) was good related to VO_2peak_ through a correlation analysis (5) (updated). In fact, CPET and 6MWT each represent an important determinant of CRF. The intensity level is a key parameter of CPET, while 6MWT places primary emphasis on walking endurance and seems to be determined more by impaired walking capacity (20, 21).

Ischemic stroke accounts for about 80% of all stroke cases (22). Among them, nearly half of them are mild in nature, which are usually defined by ≤ 5 on the National Institute of Health Stroke Scale (NIHSS) and <2 on the modified Rankin Scale (mRS) (23, 24). These patients exhibit no or mild motor dysfunction and mostly show well-preserved walking ability in the acute phase after stroke (25). However, very limited studies have investigated the CRF level in these patients with mild acute ischemic stroke. Currently, available data have shown that there is a significant decrease in the CRF level in patients with stroke from subacute to chronic phase compared to age- and sex-matched healthy control subjects. In addition, the VO_2peak_ level is gradually increasing during the subacute phase, and then trapped at a level that is about only half of the healthy control during the chronic phase (3). This leaves the question of how patients' CRF level evolves during the acute phase, and whether the CRF level is impaired in patients with mild ischemic stroke with complete motor ability. This literature gap creates a barrier for us to understand the evolution of CRF from the early stage after stroke, and to set an appropriate dose of exercise prescription. Given the characteristics of 6MWT, such as feasibility, functionality, simplicity, and low cost, we believed that 6MWT would be very suitable for investigating the CRF level in patients with mild acute ischemic stroke.

Apart from CEPT and 6MWT, many other indicators are widely used in the clinic to measure the different dimensions of CRF in patients with stroke, including 10-m walk test (10mWT), five-times sit-to-stand time (FTSST), functional reaching test (FRT), Berg Balance Scale (BBS), and grip strength (GS). Meta-analysis of cross-sectional studies reported a moderate correlation between the walking speed measured by 10mWT and VO_2peak_ (0.31 < *r* <0.54, 95%CI) (21). A prospective cohort study also revealed that the baseline maximal gait speed assessed by 10mWT in subacute stroke was positively correlated with later VO_2peak_ in the chronic phase after stroke (26). FTSST is a reliable measurement tool mainly designed for evaluating the functional lower body muscle strength (27), which is also an important component influencing stroke survivors' CRF (28). FRT and BBS both belong to the classic clinical measure of balance (29) (updated). The balance scores are not only a strong predictor of levels of locomotor activity of individuals after stroke but also related to VO_2peak_. It has been demonstrated that the BBS score correlated with the speed at the end of the CPET assessment, and patients with stroke with deficits in balance might not be able to reach sufficient walking speed to achieve an effective maximum CPET (30). GS is a simple, an inexpensive, and a useful indicator of muscular fitness, and it is strongly correlated with overall muscle strength (31). Previous studies have indicated that GS is associated with all-cause mortality, and GS itself is also a risk factor for unfavorable health outcomes in cardiovascular diseases (32). More importantly, GS is a useful prognostic biomarker for stroke and can be used to stratify an individual's risk of stroke recovery (33). Prior studies have shown that GS adjusted for the body surface area may moderately reflect CRF levels in healthy young adults (12). Furthermore, many studies observed parallel changes between the GS and CRF value in the course of the diseases like diabetes (34), liver disease (35), cardiovascular disease (36), common mental disorders (37), and cancer (38). However, to our knowledge, no prior studies have reported the level and further relationship between each other of these indicators mentioned above in patients with mild acute ischemic stroke.

To address this knowledge gap, we conducted this cross-sectional, single-time assessment study. The present study aimed to reveal the level of CRF in a group of mild ischemic stroke at the acute phase and to explore the possible association between 6MWT, GS, and other measurements related to CRF. Considering the reliability and feasibility of 6MWT in measuring CRF, we set the 6MWD as the primary indicator of CRF level and performed the correlation and regression analysis between other indicators and the 6MWD. We speculate that GS and other measurements might show a significant correlation with 6MWD to varying degrees, and 6MWD can be reflected by models consisted of the different combinations of indicators that each represent an important aspect of CRF in patients with mild acute ischemic stroke.

## Methods

### Study Population

This cross-sectional observational study enrolled 30 patients with stroke from the Department of Rehabilitation of The People's Hospital of Jiaozuo City and The Second Affiliated Hospital of Kunming Medical University. Patients were eligible if they: (1) experienced a clinically diagnosed mild ischemic stroke within 3–14 days from the onset; (2) with the NHISS (39) (updated) score ≤ 5 points; (3) were independently ambulant; (4) with no obvious motor impairments [Fugl-Meyer Motor Assessment (40) (updated) = 100]; (5) were right-handed according to the Edinburgh Handedness Inventory (EHI) (41) (updated); (6) aged between 18 and 85 years; and (7) with the mRS score (42) (updated) <2 points. Patients with any of the following were excluded: (1) cognitive dysfunction MoCA test (43) (updated) <26 points); (2) unable to complete the assessment due to deficits in hearing, vision, or understanding; (3) with severe dysfunction of one or more vital organs; and (4) with severe aphasia. This study also included 71 age- and GS-matched healthy participants from one community in Yuhang District Shanghai, China, and two communities in Xiaoshan District, Hangzhou, China. All healthy subjects were right-handed assessed by EHI, cognitively intact, and without any history of stroke or any known neurological, cardiovascular, respiratory, or musculoskeletal disease that might have affected their ability to perform the tests in our study.

Patients meeting the inclusion criteria were recruited through posters in the inpatient rehabilitation departments. Healthy subjects were screened and recruited as follows. We first calculated the mean age and GS of the stroke group. We then screened the existing data in the annual physical examination in the community and selected the matched healthy people according to the data (age and GS on the left side) of participants with stroke at the ratio of 1:3. Finally, a total of 90 healthy participants were screened and 71 were eventually recruited. The sample size was calculated based on the previously reported data and our pretest. The assumption was that the mean difference between the healthy group and the patient group with stroke would be 80 m in the 6WMD with a common SD of 120 m. The final target sample size was a total of 84–100 participants with 25–28 patients with stroke and 56–75 healthy subjects when setting a power of 80%, a significance level of 0.05, and a ratio of 1:2–1:3 between the patient group with stroke and the healthy group (44, 45).

Ethical approval for this study was obtained from the Review Board of Ethics Committee of Huashan Hospital (the ethical approval documents were then submitted to all other centers, and all participating centers did not start the recruitment until each ethics application was approved) and registered at the Chinese Clinical Trial Registry (Chi-CTR-2000031379). Written informed consent was obtained from all participants before recruitment.

### Data Collection

Participants were initially screened by an interview, then the information on demographic features (age, sex, height, and weight), degree of neurologic deficits, stroke history, and cognition was collected *via* a series of structured questionnaires. All recruited patients and healthy subjects underwent a single-time assessment of indicators related to CRF, including 6MWT, GS of left hand (GS-left), GS of right hand (GS-right), 10mWT, FTSST, FRT, mRS, waistline, and BBS. All assessments were performed by four well-trained physiotherapists, one in each research center (one of the therapists was responsible for data collection in two adjacent communities in Xiaoshan District, Hangzhou, China), according to the standardized evaluator manual.

### 6-Min Walk Test

**Figure 1:**
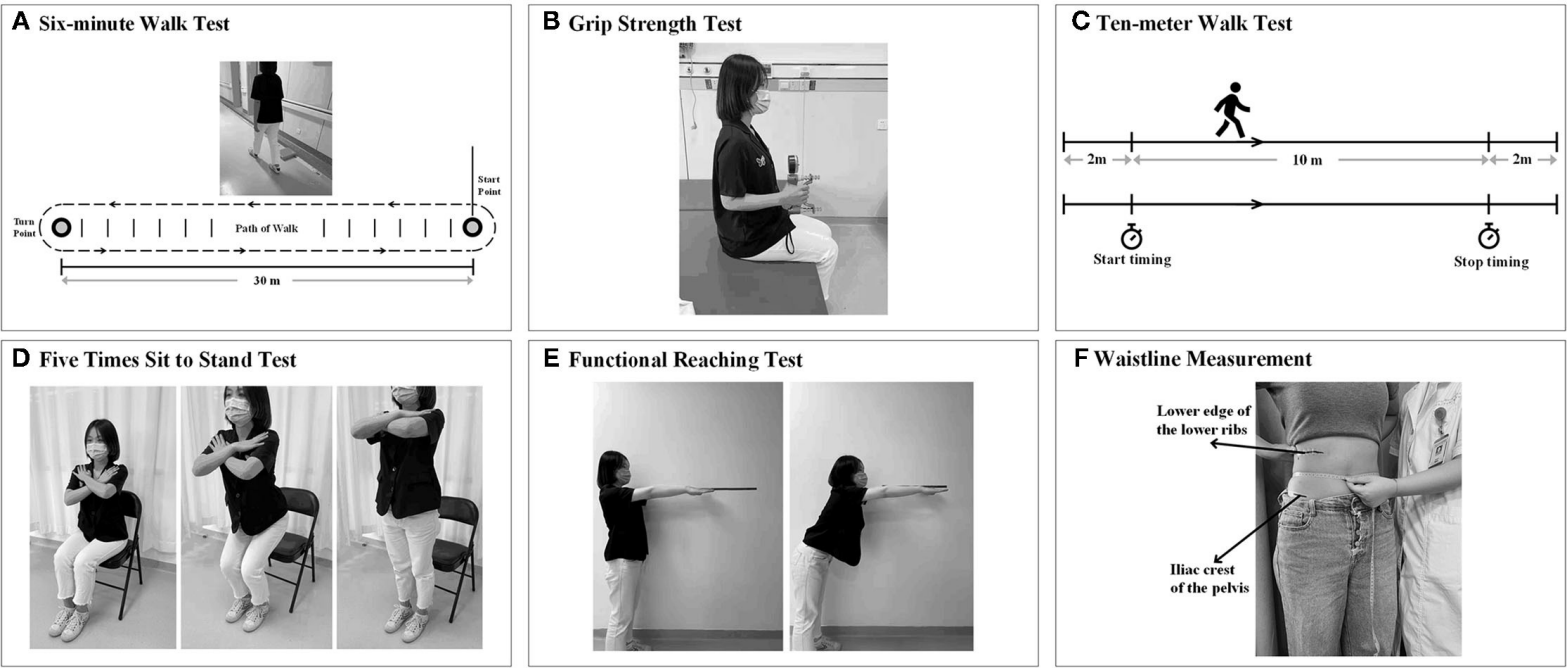
Measurement of indicators related to cardiorespiratory fitness (CRF). **(A)** 6-min walk test (6MWT). **(B)** Grip strength (GS) test. **(C)** 10-m walk test (10 mWT). **(D)** The five-times sit-to-stand test. **(E)** Functional reaching test (FRT). **(F)** Waistline measurement (circumference at the middle of the lower edge of the lower ribs and the iliac crests of the pelvis).

The 6MWT (m) is a well-validated, easy-to-administer submaximal exercise test (46). The 6MWD assessed from 6MWT was used as the primary indicator of CRF in this study (16). Patients with stroke and healthy subjects were instructed to walk as far as possible along a 30-m, continuous track with a hard surface, turn around a marker cone, walk in the opposite direction, and repeat this loop during a 6-min period. The linear track had a long horizontal line at both ends, with a short horizontal line placed every 3 m along the course. Ankle braces, walkers, canes, and other assistive devices for walking were not used in our study because all patients had an Fugl-Meyer assessment (FMA) score of 100 (Figure 1A). Participants were permitted to rest during the test if they felt exhausted but were instructed to resume walking as soon as they could. Moreover, verbal encouragement was provided during the measurements every minute after each minute.

### GS Test

Grip strength (kg) was measured using a hydraulic hand dynamometer (Jamar J00105, Exacta, North Coast Medical Inc., Gilroy, CA, USA) following a standardized measurement process according to a previous study (47). The participants were seated upright with their shoulder adducted and neutrally rotated, elbow flexed at 90°, forearm facing forward, and resting on an armrest. The participants were instructed to complete a 3-s maximal isometric handgrip effort three times, expressed as kilograms (Figure 1B). The average value of the three times on each side (GS-left and GS-right) was calculated and used in the analysis. Verbal encouragement was given to the subjects during the GS test. The average value of the three times on each side (GS-left and GS-right) was calculated and used in the analysis.

### 10-M Walk Test

The 10mWT was performed to obtain the maximal walking speed when the participants walked for the middle 10-m section of a 14-m straight corridor. The first 2 m and the last 2 m were used for acceleration and deceleration, respectively. According, timing started and stopped when the participant's first foot crossed the 2-m mark and the 12-m mark (Figure 1C). Encouragement was not given during the whole test (46). The walking speed was computed as the average velocity during the middle 10-m section (i.e., meters/second).

### Five-Times Sit-to-Stand Test

The FTSST is a quick and convenient test to evaluate mainly the functional lower extremity strength and balance of an individual (48). The participants were seated with their arms folded across their chest, and with their hips and knees at approximately 90° and 105° of flexion, respectively, at the beginning of the test (Figure 1D). The time (in seconds) that participants complete five repetitions of the sit-to-stand maneuver from an armless chair at a maximum and safe speed is measured (49).

### Functional Reaching Test

The FRT(s) was used to assess the limit of stability by measuring the maximum distance which the participants can reach forward while standing in a fixed position. The participants were required to stand next to but not touch a wall with their feet shoulder-width apart, and to position the arm at 90° of shoulder flexion with a closed fist (50). After recording the third metacarpal head as the first point on the yardstick, the assessors instructed the participants to reach forward as far as possible without taking a step and recorded the last point. The participants should maintain the shoulder height and the upper limb level for at least 5 s without losing balance (Figure 1E). The scores were noted as the difference in the distance between the first and last point. The test will be completed three times, and the average of the last two was determined as the limit of stability (51).

### Berg Balance Scale

The BBS is a functional balance scale consisting of 14 functional balance tasks (52). It assessed both static balance and dynamic balance by asking the participants to perform a series of tasks in different postures (Figure 1F). Each item on the BBS scale was rated from 0 to 4, and the maximum score was 56. The intra- and inter-rater reliability, as well as sensitivity to change of BBS, were excellent, and this scale has been confirmed to be an effective way of balance measurement in patients with stroke (53).

### Waistline Measurement

Previous studies have suggested the inverse relationship between waist circumference and CRF (54, 55). The subject was asked to remain in a standing position, after which the waist between the lowest rib edge and the iliac crest was measured. We used a flexible tape measure to measure the waist to the nearest 0.1 cm (54).

### Statistical Analysis

Statistical analyses were conducted using SPSS 25.0 (IBM Corporation, Armonk, NY, USA). Continuous data are presented as the mean ± SD, and frequencies were calculated for categorical variables. The normality of data distribution and residuals from linear regressions were evaluated using the Shapiro–Wilk normality test. The differences between the groups were evaluated using the independent samples *t*-tests. Non-parametrical tests were conducted when the values did not follow a normal distribution. A correlation analysis between 6MWD and other indicators related to CRF was performed using the Pearson correlation analysis in both groups, respectively. We defined *r* > 0.6 as a strong correlation, 0.3 < *r* <0.6 as a moderate correlation, and *r* <0.3 as a weak correlation. All indicators, which demonstrated significant moderate or higher correlations (*p* < 0.01, *r* > 0.3) with 6WMD, were entered into stepwise multiple linear regression analysis to evaluate their potential values in predicting CRF as measured by 6MWD in each group. Multicollinearity (strong correlations among independent variables) was examined by collinearity diagnostic statistics. The value of *p* < 5% was considered significant in this study with symbols presenting as ^*^ for *p* < 0.05, ^**^ for *p* < 0.01, and ^***^for *p* < 0.001.

## Results

Data from 30 patients with mild acute ischemic stroke [8F/22M, time since stroke onset = 8.2 ± 2.9 days, affected side: 14-left/7-right/9-bilateral, location of lesion: 13-cortical/7-subcortical/2-cerebellar/1-brainstem/7-multiple, NIHSS = 2.00 ± 1.82, MRS = 0.83 ± 0.38] and 71 healthy control subjects [38F/33M] were included for data analysis. All recruited participants successfully underwent the whole procedure of interview and assessment, and none of them withdrew from the study. The details of demographic and indicators related to CRF of patients with stroke and age- and GS-matched healthy subjects are displayed in Table 1 and Supplementary Figure S1.

**Table 1:** Demographic and clinical characteristics related to cardiorespiratory fitness (CRF) of the two groups.

	**Patients with stroke**	**Healthy subjects**	***P*-value**
	***N* = 30**	***N* = 71**	
Age (years)	59.8 ± 9.4	57.9 ± 9.5	0.131
BMI (kg/m^2^)	25.4 ± 3.1	23.5 ± 2.6	0.002
Sex (F/M)	8/22	38/33	0.016
Waistline (cm)	94.3 ± 9.3	81.9 ± 8.0	0.000
GS-left (kg)	28.63 ± 10.15	29.37 ± 12.96	0.885
GS-right (kg)	29.67 ± 10.59	31.37 ± 14.37	0.967
FTSST (s)	14.41 ± 4.11	9.14 ± 2.01	0.000
FRT (cm)	24.3 ± 7.5	34.5 ± 8.1	0.000
BBS	50.73 ± 4.96	54.86 ± 4.46	0.000
10mWT (m/s)	1.46 ± 0.39	1.35 ± 0.32	0.059
6MWD (m)	419.7 ± 82.4	459.5 ± 81.8	0.029

*BMI, body mass index; GS, grip strength; FTSST, five-times sit-to-stand test; FRT, functional reaching test; BBS, Berg Balance Scale; 10mWT, 10-m walk test; 6MWD, 6-min walk distance*.

### Group Comparisons Between Patients With Stroke and Healthy Subjects

As shown in Supplementary Figure S1, the group of patients with mild acute ischemic stroke and the healthy control group were matched in age, GS-left, and GS-right. The patient group showed significantly higher values of body mass index (BMI) (*t* = 3.190, *p* = 0.002) and waistline (*t* = 6.732, *p* < 0.001) than the healthy group. In addition, there were significant differences in FTSST, FRT, BBS, and 6WMD between the two groups. The stroke group had a significantly higher FTSST score (*t* = 8.679, *p* < 0.001), while it had a significantly lower score of FRT (*t* = 5.550, *p* < 0.001), BBS (*t* = 4.111, *p* < 0.001), and 6WMD (*t* = 3.190, *p* = 0.002). However, there was no statistical difference between the two groups in 10mWT (*t* = 2.230, *p* = 0.028).

### Association Between 6MWD and Other CRF-Related Indicators

#### Mild Acute Ischemic Stroke Group

**Figure 2:**
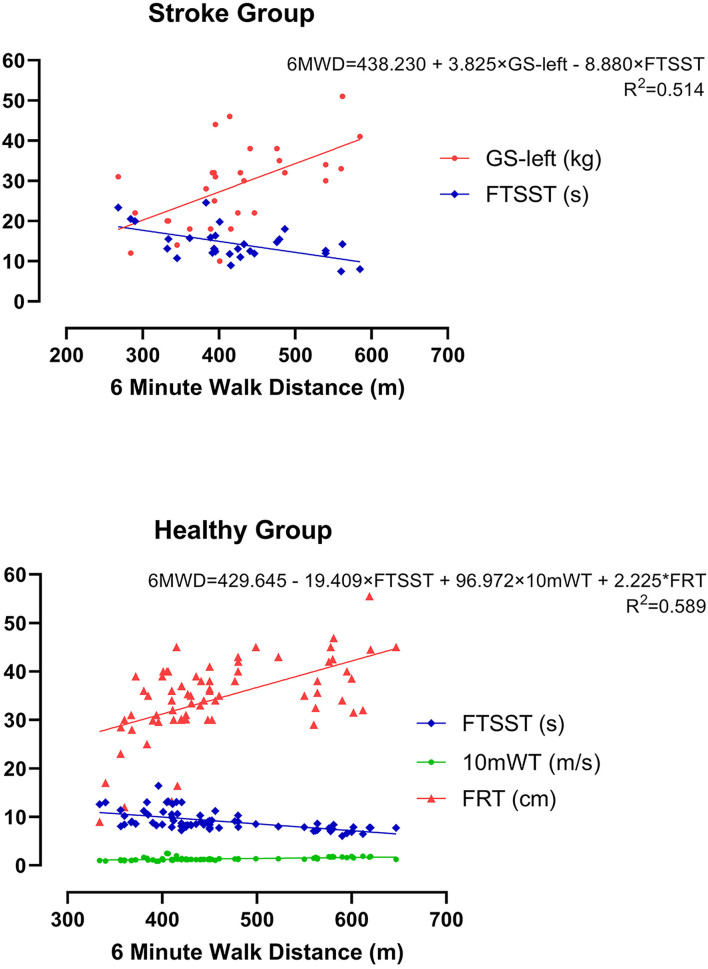
Correlation matrix of the stroke group. NIHSS, National Institutes of Health Stroke Scale; MRS, modified Rankin Scale; GS, grip strength; BMI, body mass index; FTSST, five-times sit-to-stand test; FRT, functional reaching test; BBS, Berg Balance Scale; 10mWT, 10-m walk test; 6MWD, 6-min walk distance.

The results of Pearsons' Correlation Coefficient analysis between 6MWD and other variables related to CRF are shown as a correlation matrix in Figure 2. Significant positive correlations were found between 6MWD and GS-left (*r* = 0.573, *p* = 0.001), GS-right (*r* = 0.524, *p* = 0.003), 10mWT (*r* = 0.554, *p* = 0.001), and FRT (*r* = 0.449, *p* = 0.021), showing that better GS, 10mWT, and FRT correlated with a higher CRF as measured by 6MWT in the stroke group. In addition, a significant negative correlation was found between 6MWD and FTSST (*r* = −0.551, *p* = 0.002), suggesting that better functional lower extremity strength and balance were associated with a higher CRF. There were no correlations between 6MWD and other variables.

Subsequently, these candidates' CRF-related indicators (GS-left, GS-right, 10mWT, and FTSST), which demonstrated significant moderate correlations (*p* < 0.01, *r* > 0.3) with 6WMD, were entered into stepwise multiple linear regression analysis. As illustrated in Table 2, two different models were generated, and GS-left was an independent predictor of the current CRF level as measured by 6MWT in both models. We chose the second model as the optimum model, which included two indicators and manifested as 6MWT = 438.230+3.825^*^GS-left-8.880^*^FTSST (*r*^2^ = 0.514) (**Figure 4A**).

**Table 2:** The result of multiple linear regression analysis coefficients in the stroke group^a^.

		**Unstandardized** **coefficients**		**Standardized** **coefficients**	***t***	***P***	***r* ^2^**	**Collinearity statistics**	
**Model**	**Predictors**	***b***	***SE***	**β**				**Tolerance**	**VIF**
1	Constant	286.495	38.147		7.510	<0.001	0.328		
	GS-left (kg)	4.652	1.258	0.573	3.698	0.001		1.000	1.000
2	Constant	438.230	57.633		7.604	<0.001	0.514		
	GS-left (kg)	3.825	1.120	0.471	3.416	0.002		0.947	1.056
	FTSST (s)	−8.880	2.764	−0.443	−3.213	0.003		0.947	1.056

a *Dependent variable: 6MWT.*

*VIF, variance inflation factor*.

#### Healthy Control Group

**Figure 3:**
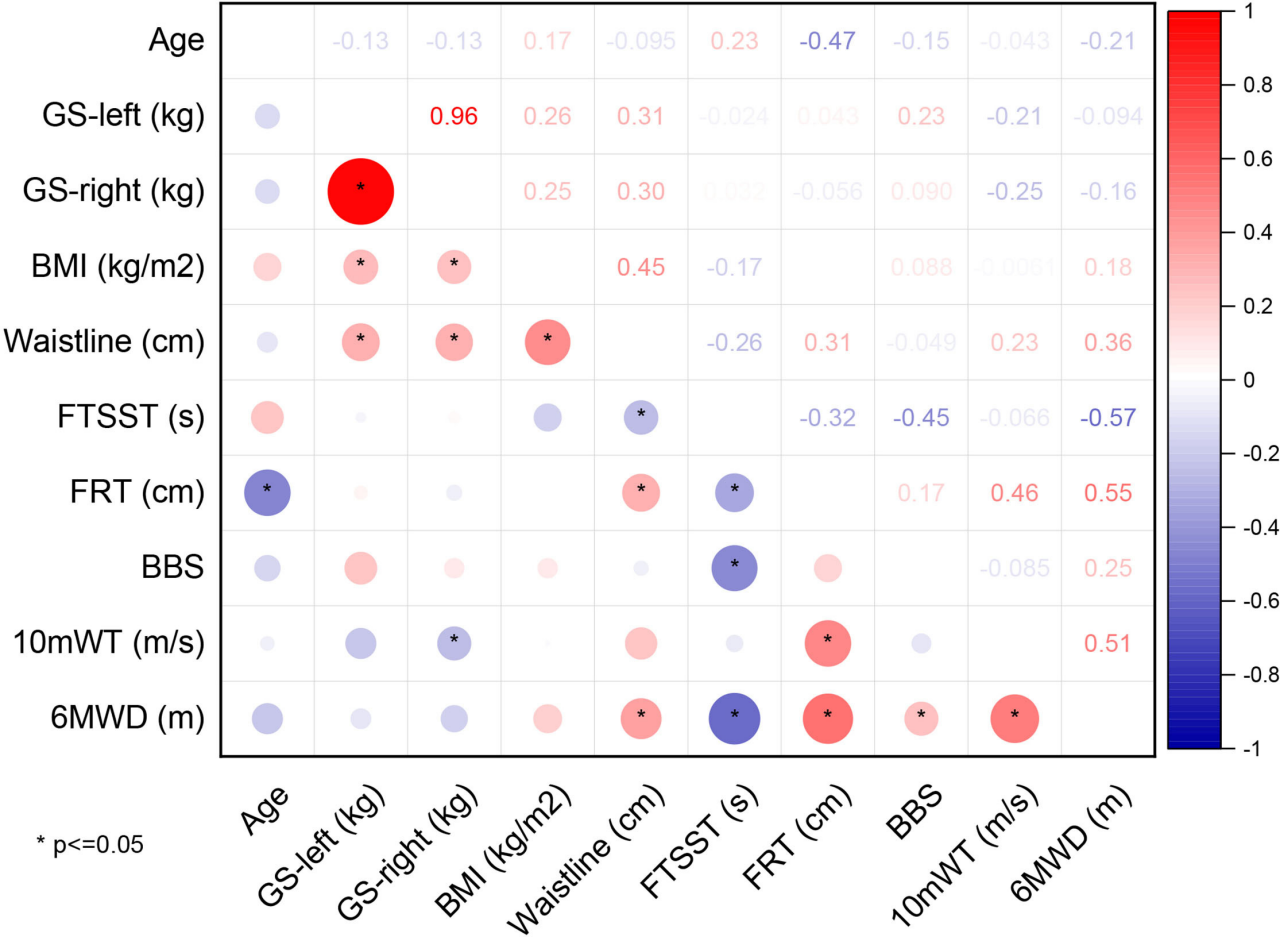
Correlation matrix of the healthy group. GS, grip strength; BMI, body mass index; FTSST, five-times sit-to-stand test; FRT, functional reaching test; BBS, Berg Balance Scale; 10mWT, 10-m walk test; 6MWD, 6-min walk distance.

The correlations between 6MWD and other CRF-related variables are shown as a correlation matrix in Figure 3. Significant positive correlations were found between 6MWD and waistline (*r* = 0.364, *p* = 0.001), 10mWT (*r* = 0.512, *p* = 0.003), FRT (*r* = 0.550, *p* < 0.001), and BBS (*r* = 0.248, *p* = 0.021), showing that larger waistline as well as better 10mWT, FRT, and BBS implied a higher CRF in healthy subjects. Similar to the stroke group, a significant negative correlation was found between 6MWD and FTSST (*r* = −0.573, *p* = 0.002) in the healthy control group. There were no correlations between 6MWD and other variables.

**Figure 4:**
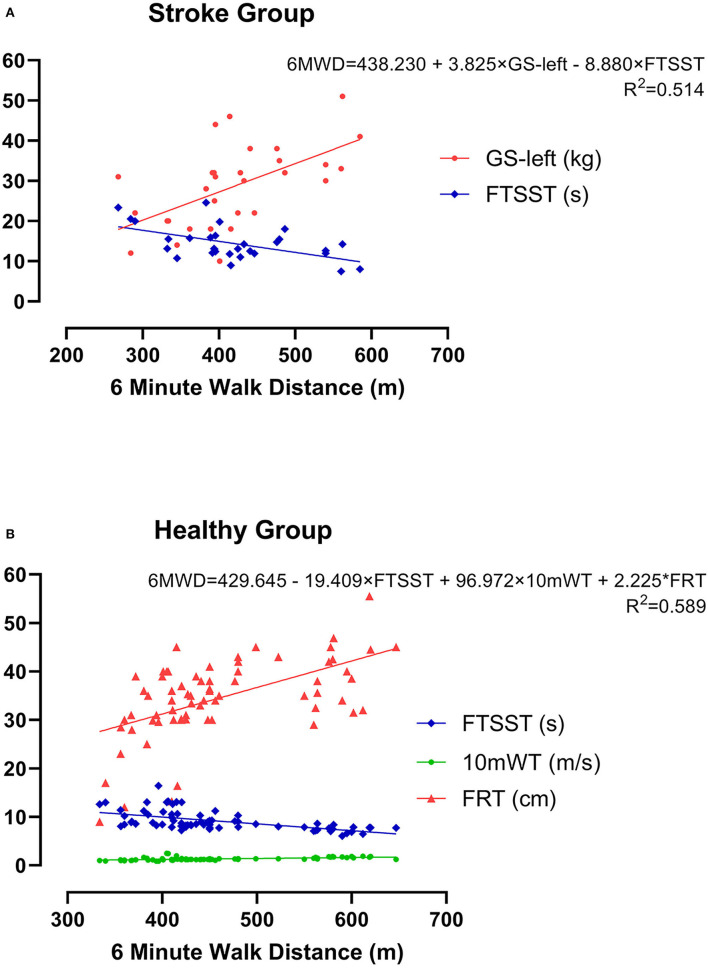
The optimum model for estimating CRF as measured by 6MWD using multiple linear regression analysis in both groups. **(A)** Mild acute ischemic stroke group and **(B)** healthy control group.

Likewise, the candidate CRF-related indicators (waistline, 10mWT, FRT, and FTSST), which showed significant moderate correlations (*p* < 0.01, *r* > 0.3) with 6WMD, were entered into stepwise multiple linear regression analysis. As is shown in Table 3, three different models were generated. We chose the third model, which showed the largest *r*^2^ as the optimum model. It included three indicators and manifested as 6MWT = 429.645-19.409^*^FTSST+96.972^*^10mWT+2.25^*^FRT (*r*^2^ = 0.589) (Figure 4B).

**Table 3:** The result of multiple linear regression analysis coefficients in the healthy group^a^.

		**Unstandardized**		**Standardized**	***t***	***P***	***r* ^2^**	**Collinearity**	
		**coefficients**		**coefficients**				**statistics**	
**Model**	**Predictors**	***b***	***SE***	**β**				**Tolerance**	**VIF**
1	Constant	672.553	37.538		17.917	<0.001	0.328		
	FTSST (s)	−23.316	4.014	−0.573	−5.809	<0.001		1.000	1.000
2	Constant	496.499	42.965		11.556	<0.001	0.555		
	FTSST (s)	−22.042	3.300	−0.542	−6.680	<0.001		0.996	1.004
	10mWT (m/s)	122.153	20.788	0.477	5.876	<0.001		0.996	1.004
3	Constant	429.645	50.295		8.543	<0.001	0.589		
	FTSST (s)	−19.409	3.383	−0.477	−5.738	<0.001		0.888	1.127
	10mWT (m/s)	96.972	22.768	0.378	4.259	<0.001		0.778	1.286
	FRT (cm)	2.225	0.941	0.221	2.363	0.021		0.700	1.428

a *Dependent Variable: 6MWT.*

*VIF, variance inflation factor*.

## Discussion

In this study, we performed a series of measurements related to CRF in a group of patients with mild ischemic stroke at the acute phase and a group of age- and GS-matched healthy subjects. To our knowledge, this is the first study looking into the status of the CRF level in patients with mild ischemic stroke at the acute phase. Overall, we made three levels of observation. First, we compared the demographic and clinical characteristics related to CRF between the two groups. The comparison results of CRF-related variables suggested that patients with mild ischemic stroke at the acute phase did show a lower level in CRF even though we only recruited patients with no evidence of neurologic deficits. Second, the correlation analysis showed that quite a few variables were significantly associated with CRF value as measured by 6MWT in both groups. We also found that the patient group with stroke and the healthy group demonstrated the two distinct patterns of a correlation matrix, implying that the different dimensions of CRF were distributed differently in each group. Third, a multilinear regression analysis demonstrated that GS-left and FTSST were the indicators of CRF in the patient group with stroke, while FTSST, 10mWT, and FRT can partially represent the CRF level in the healthy group.

In this study, the healthy group had significantly higher scores in FRT, BBS, and 6WMD, and a significantly lower FTSST than the patient group with stroke. These results have provided direct evidence to the viewpoint that the CRF level has already declined in patients with mild ischemic stroke within 2 weeks after stroke as we have set the 6MWD as the primary indicator of CRF. It is worth mentioning that we only recruited patients with stroke having the NHISS score ≤ 5 points and with mRS score <2 points, which indicated that they did not have apparent functional impairments. Because of this, the problem of CRF loss in these patients with mild stroke might be easily overlooked, thus we could hardly find any published literature reporting the data about the change of CRF in patients with mild stroke at the acute phase. In fact, the real difference of 6MWD between patients with mild ischemic stroke at the acute phase and healthy people should be slightly greater than the current difference in this study. This is because the participants in the two groups were matched for GS (both left and right). For the GS, the mean value in the stroke group was about 29 kg and the mean value in the healthy group was about 30 kg, both of which were slightly lower than the normal value of 60 years old (55, 56). Moreover, the average value of 6MWD in both groups was shorter than the reference value, which is more than 500 m for people around the age of 60 years old (57, 58). Thus, if this study recruited a group of healthy control naturally without considering the matching of GS, the difference of CRF level between patients with mild ischemic stroke at the acute phase and healthy control people should have been further expanded. All these analyses point to a reasonable speculation that the CRF level indeed significantly decreases in patients with mild ischemic stroke at the acute phase. Admittedly, this speculation should be verified by measuring VO_2peck_ through CPET in further studies. Though the associations between 6MWD and VO_2peak_ of patients with stroke range from 0.29 to 0.74, one meta-analyses including 13 studies involving 454 patients with stroke showed combined correlation coefficients for VO_2peak_ and walking distance of 0.52 (95% credibility interval = 0.42, 0.62) (21). More importantly, previous studies have shown that the 6MWD was good related to VO_2peak_ through a correlation analysis (5). In any case, the finding of the lower level of CRF in patients with mild ischemic stroke at the acute phase has meaningful clinical implications. It is known that walking disability (59), poor cognitive performance (60), and limitations in activities of daily living (5) may cause CRF reduction, which causes further deconditioning (61). Therefore, it is crucial to assess the CRF in patients with mild ischemic stroke early after stroke onset to develop appropriate therapeutic strategies to prevent CRF from decreasing or facilitate CRF recovery. Meanwhile, the underlying mechanism for the lower level of CRF shortly after stroke is worth digging out in future research.

Additional results of comparison between the groups are very intriguing as well. Previous studies showed that both waistline and BMI are the indicators of obesity, which is associated with an increased risk of ischemic stroke (62). In this study, the average waistline of patients with stroke was greater than healthy subjects, but the BMI of the two groups has no significant changes. This finding was consistent with previous research that waistline could act as a better predictor for obesity-related comorbidities than BMI (63). Thus, it would be important to control body weight and to monitor risk factors in overweight people to reduce the odds of stroke. Another interesting phenomenon was that there was no statistical difference between the two groups in 10mWT. Even though 10mWT is closely related to CRF in patients with subacute and chronic stroke (21), it focuses more on the explosive strength and motor control of the lower extremity. Because 10mWT has a relatively short distance (26), which means it might be difficult to push the exercise load to the cardiorespiratory limit in patients with mild ischemic stroke at the acute phase.

As for the results of a correlation analysis, our results showed that several variables related to CRF were associated with 6MWD in two groups, but the two groups demonstrated two distinct patterns of correlation matrix. Specifically, significant positive correlations were found between 6MWD and GS-left, GS-right, FRT, and 10mWT in the stroke group, and between 6MWD and waistline, 10mWT, FRT, and BBS in the healthy group. Moreover, a significant negative correlation was found between 6MWD and FTSST in both groups. This result once again suggested that all CRF-related variables selected in this study were closely related to the CRF level as measured by 6MWT. Whereas, the difference in a correlation matrix between the group implied that the different dimensions of CRF (i.e., strength, balance, endurance, etc.) were distributed differently in each group, and the ability of each variable to predict CRF was not the same in different groups of people.

Stepwise multiple linear regression analysis furthers revealed that GS-left and FTSST were associated with 6MWD in patients with mild ischemic stroke at the acute phase, while FTSST, 10mWT, and FRT were associated with 6MWD in healthy subjects. GS is commonly used as a simple, an inexpensive, and a useful indicator of muscular fitness. But so far, few studies have investigated its value in predicting CRF (12). Although both GS-left and GS-right were correlated with 6MWD in patients with stroke, only GS-left was selected into the optimum model for estimating CRF after multiple linear regression analyses. These findings confirm GS-left as a potential indicator of CRF of patients with stroke. In other words, for the patients who cannot perform CPET or 6MWT, GS-left is an easy-to-use alternative way to evaluate their CRF. For the human, handedness can affect brain functional and structural organizations (64, 65). In this study, all patients with stroke were right-handed. We speculate that the GS-right is largely affected by the frequency and intensity of individual use in life and work. The use frequency and intensity of the left hand are closer to the lower limb, trunk, cardiopulmonary system, or other tissues and organs of the body, so GS-left might better represent the level of CRF in patients with mild ischemic stroke at the acute phase. More work is needed to explore the mechanisms underlying this phenomenon. In our study, there was no significant correlation between GS and CRF in the healthy group. This result is inconsistent with a previous study, which found that GS on the dominant side could moderately reflect CRF levels in healthy young adults (12). The inconsistencies between our results and this previous study might be explained by the reason that VO_2peak_ was used as the index for CRF, while we used 6MWT. In future studies, we should use VO_2peak_ to further verify the relationship between CRF and GS.

The FTSST, which is a valid measure of balance performance in people with stroke (66), was longer in the patient group than the healthy group in our study. Combined with the result that FTSST could reflect the CRF level in both groups, we suppose that the lower level of CRF in the stroke group might be related to impaired balance ability in patients though they could move without restraint. Also, other indicators related to CRF, including FRT and BBS were worse in patients with stroke. FRT and BBS are widely used as assessment tools for both static and dynamic balance in clinical settings (67). The balance represents the ability to control the center of mass concerning the base of support (68). It is well-known that many factors could influence human balance, such as central (69), sensory input, muscle strength, and range of motion. The findings of this study mean that balance plays an important role in a 6WMT for both patients with stroke and healthy subjects. At the same time, FTSST and FRT may share a much closer relationship to 6MWT than BBS. In addition, GS was also found to correlate significantly with lower limb gait and balance measures in a previous study (70), which indicated a potential relationship between upper and lower extremities.

Several limitations exist in our study. First, the sample size of the stroke group was relatively small. Future studies with larger sample sizes are necessary to confirm the findings of this study. Second, we focus only on patients with ischemic stroke at the acute phase with an intact function (mRS <2). In future studies, subjects with different levels of functional status should be recruited and studied. In addition, future studies are needed to verify our findings using CPET as the gold standard for CRF measurement.

## Conclusions

Returning to the question posed at the beginning of this study, it is now possible to state that the CRF is impaired in patients with mild ischemic stroke at the acute phase. Moreover, GS-left has a positive relationship with CRF measured by 6WMD and may be a complementary method for CPET to assess CRF in patients with stroke.

## Data Availability Statement

The original contributions presented in the study are included in the article/Supplementary Material, further inquiries can be directed to the corresponding author/s.

## Ethics Statement

The studies involving human participants were reviewed and approved by the Review Board of the Ethics Committee of Huashan Hospital (KY2020-017). The patients/participants provided their written informed consent to participate in this study. Written informed consent was obtained from the individual(s) for the publication of any potentially identifiable images or data included in this article.

## Author Contributions

QQ and JZ contributed to the data collection, statistical analysis, result interpretation, and manuscript writing. HW contributed to the data collection and statistical analysis. QF, YZ, ZY, and QZ contributed to the data collection. YW, CZ, YH, and LY also participated in this study. JJ contributed to the conception and revision of the manuscript. All authors contributed to the article and approved the submitted version.

## Funding

This study was supported by the National Key Research & Development Program of the Ministry of Science and Technology of the People's Republic of China (Grant Nos. 2018YFC2002300 and 2018YFC2002301), the National Natural Science Foundation of China (Grant No. 91948302), the China National Nature Science Young Foundation (Grant No. 82102665), the Shanghai Sailing Program (Grant No. 21YF1404600), and the Innovative Research Group Project of National Natural Science Foundation of China (Grant No. 82021002).

## Conflict of Interest

The authors declare that the research was conducted in the absence of any commercial or financial relationships that could be construed as a potential conflict of interest.

## Publisher's Note

All claims expressed in this article are solely those of the authors and do not necessarily represent those of their affiliated organizations, or those of the publisher, the editors and the reviewers. Any product that may be evaluated in this article, or claim that may be made by its manufacturer, is not guaranteed or endorsed by the publisher.

## References

[1] Wang W Jiang B Sun H Ru X Sun D Wang L. Prevalence, incidence, and mortality of stroke in china: results from a nationwide population-based survey of 480 687 adults. Circulation. (2017) 135:759–71. 10.1161/CIRCULATIONAHA.116.025250 28052979

[2] Kodama S Saito K Tanaka S Maki M Yachi Y Asumi M. Cardiorespiratory fitness as a quantitative predictor of all-cause mortality and cardiovascular events in healthy men and women: a meta-analysis. JAMA. (2009) 301:2024–35. 10.1001/jama.2009.681 19454641

[3] Fan Q Jia J. Translating research into clinical practice: importance of improving cardiorespiratory fitness in stroke population. Stroke. (2020) 51:361–7. 10.1161/STROKEAHA.119.027345 31813356

[4] Outermans JC van de Port I Kwakkel G Visser-Meily JM Wittink H. The role of postural control in the association between aerobic capacity and walking capacity in chronic stroke: a cross-sectional analysis. Eur J Phys Rehabil Med. (2018) 54:837–44. 10.23736/S1973-9087.18.04987-0 29532648

[5] Kim BR Han EY Joo SJ Kim SY Yoon HM. Cardiovascular fitness as a predictor of functional recovery in subacute stroke patients. Disabil Rehabil. (2014) 36:227–31. 10.3109/09638288.2013.787123 23594057

[6] Hafez S Eid Z Alabasi S Darwiche Y Channaoui S Hess DC. Mechanisms of preconditioning exercise-induced neurovascular protection in stroke. J Stroke. (2021) 23:312–26. 10.5853/jos.2020.03006 34649377 PMC8521252

[7] Fini NA Holland AE Keating J Simek J Bernhardt J. How is physical activity monitored in people following stroke? Disabil Rehabil. (2015) 37:1717–31. 10.3109/09638288.2014.978508 25374044

[8] Johnson L Kramer SF Catanzariti G Kaffenberger T Cumming T Bernhardt J. Safety of performing a graded exercise test early after stroke and transient ischemic attack. PM R. (2020) 12:445–53. 10.1002/pmrj.12259 31600415

[9] Smith AC Saunders DH Mead G. Cardiorespiratory fitness after stroke: a systematic review. Int J Stroke. (2012) 7:499–510. 10.1111/j.1747-4949.2012.00791.x 22568786

[10] Wang H Xu G Wang X Sun C Zhu B Fan M. The reorganization of resting-state brain networks associated with motor imagery training in chronic stroke patients. IEEE Trans Neural Syst Rehabil Eng. (2019) 27:2237–45. 10.1109/TNSRE.2019.2940980 31536007

[11] McCain EM Dick TJM Giest TN Nuckols RW Lewek MD Saul KR. Mechanics and energetics of post-stroke walking aided by a powered ankle exoskeleton with speed-adaptive myoelectric control. J Neuroeng Rehabil. (2019) 16:57. 10.1186/s12984-019-0523-y 31092269 PMC6521500

[12] Zhou M Zha F Chen Y Liu F Zhou J Long J. Handgrip strength-related factors affecting health outcomes in young adults: association with cardiorespiratory fitness. Biomed Res Int. (2021) 2021:6645252. 10.1155/2021/6645252 33969122 PMC8084643

[13] Grace SL Turk-Adawi KI Contractor A Atrey A Campbell NR Derman W. Cardiac rehabilitation delivery model for low-resource settings: an international council of cardiovascular prevention and rehabilitation consensus statement. Prog Cardiovasc Dis. (2016) 59:303–22. 10.1016/j.pcad.2016.08.004 27542575

[14] Stoller O de Bruin ED Schindelholz M Schuster-Amft C de Bie RA Hunt KJ. Cardiopulmonary exercise testing early after stroke using feedback-controlled robotics-assisted treadmill exercise: test-retest reliability and repeatability. J Neuroeng Rehabil. (2014) 11:145. 10.1186/1743-0003-11-145 25306061 PMC4271449

[15] Billinger SA Arena R Bernhardt J Eng JJ Franklin BA Johnson CM. Physical activity and exercise recommendations for stroke survivors: a statement for healthcare professionals from the american heart association/american stroke association. Stroke. (2014) 45:2532–53. 10.1161/STR.0000000000000022 24846875

[16] Dunn A Marsden DL Barker D van Vliet P Spratt NJ Callister R. Evaluation of three measures of cardiorespiratory fitness in independently ambulant stroke survivors. Physiother Theory Pract. (2019) 35:622–32. 10.1080/09593985.2018.1457746 29601228

[17] Eng JJ Dawson AS Chu KS. Submaximal exercise in persons with stroke: test-retest reliability and concurrent validity with maximal oxygen consumption. Arch Phys Med Rehabil. (2004) 85:113–8. 10.1016/S0003-9993(03)00436-2 14970978 PMC3167868

[18] Mehrholz J Wagner K Rutte K Meissner D Pohl M. Predictive validity and responsiveness of the functional ambulation category in hemiparetic patients after stroke. Arch Phys Med Rehabil. (2007) 88:1314–9. 10.1016/j.apmr.2007.06.764 17908575

[19] Agarwala P Salzman SH. Six-minute walk test: clinical role, technique, coding, and reimbursement. Chest. (2020) 157:603–11. 10.1016/j.chest.2019.10.014 31689414 PMC7609960

[20] Awad LN Reisman DS Wright TR Roos MA Binder-Macleod SA. Maximum walking speed is a key determinant of long distance walking function after stroke. Top Stroke Rehabil. (2014) 21:502–9. 10.1310/tsr2106-502 25467398 PMC4382083

[21] Outermans J van de Port I Wittink H de Groot J Kwakkel G. How strongly is aerobic capacity correlated with walking speed and distance after stroke? Systematic review and meta-analysis. Phys Ther. (2015) 95:835–53. 10.2522/ptj.20140081 25573761

[22] Mozaffarian D Benjamin EJ Go AS Arnett DK Blaha MJ Cushman M. Heart disease and stroke statistics-2016 update: a report from the american heart association. Circulation. (2016) 133:e38–360. 10.1161/CIR.0000000000000350 26673558

[23] Saber H Khatibi K Szeder V Tateshima S Colby GP Nour M. Reperfusion therapy frequency and outcomes in mild ischemic stroke in the united states. Stroke. (2020) 51:3241–9. 10.1161/STROKEAHA.120.030898 33081604

[24] Terrill AL Schwartz JK Belagaje SR. Best practices for the interdisciplinary rehabilitation team: a review of mental health issues in mild stroke survivors. Stroke Res Treat. (2018) 2018:6187328. 10.1155/2018/6187328 29973980 PMC6008610

[25] Langhorne P Collier JM Bate PJ Thuy MN Bernhardt J. Very early versus delayed mobilisation after stroke. Cochrane Database Syst Rev. (2018) 10:D6187. 10.1002/14651858.CD006187.pub3 30321906 PMC6517132

[26] Gunnes M Aksetøy IA Follestad T Indredavik B Askim T. Can functional walk tests add value to the prediction of cardiorespiratory fitness after stroke? A prospective cohort study. PLoS One. (2021) 16:e255308. 10.1371/journal.pone.0255308 34339475 PMC8328339

[27] Mong Y Teo TW Ng SS. 5-repetition sit-to-stand test in subjects with chronic stroke: reliability and validity. Arch Phys Med Rehabil. (2010) 91:407–13. 10.1016/j.apmr.2009.10.030 20298832

[28] Regan EW Handlery R Stewart JC Pearson JL Wilcox S Fritz S. Integrating survivors of stroke into exercise-based cardiac rehabilitation improves endurance and functional strength. J Am Heart Assoc. (2021) 10:e17907. 10.1161/JAHA.120.017907 33499647 PMC7955427

[29] Alenazi AM Alshehri MM Alothman S Rucker J Dunning K D'Silva LJ. Functional reach, depression scores, and number of medications are associated with number of falls in people with chronic stroke. PM R. (2018) 10:806–16. 10.1016/j.pmrj.2017.12.005 29288141 PMC7200172

[30] Ovando AC Michaelsen SM Carvalho T Herber V. Evaluation of cardiopulmonary fitness in individuals with hemiparesis after cerebrovascular accident. Arq Bras Cardiol. (2011) 96:140–7. 10.1590/S0066-782X2011005000001 21448510

[31] Roberts HC Denison HJ Martin HJ Patel HP Syddall H Cooper C. A review of the measurement of grip strength in clinical and epidemiological studies: towards a standardised approach. Age Ageing. (2011) 40:423–9. 10.1093/ageing/afr051 21624928

[32] Wu Y Wang W Liu T Zhang D. Association of grip strength with risk of all-cause mortality, cardiovascular diseases, and cancer in community-dwelling populations: a meta-analysis of prospective cohort studies. J Am Med Dir Assoc. (2017) 18:517–51. 10.1016/j.jamda.2017.03.011 28549705

[33] Liu G Xue Y Wang S Zhang Y Geng Q. Association between hand grip strength and stroke in china: a prospective cohort study. Aging. (2021) 13:8204–13. 10.18632/aging.202630 33686964 PMC8034956

[34] Tarp J Støle AP Blond K Grøntved A. Cardiorespiratory fitness, muscular strength and risk of type 2 diabetes: a systematic review and meta-analysis. Diabetologia. (2019) 62:1129–42. 10.1007/s00125-019-4867-4 31011778 PMC6560020

[35] Hao L Wang Z Wang Y Wang J Zeng Z. Association between cardiorespiratory fitness, relative grip strength with non-alcoholic fatty liver disease. Med Sci Monit. (2020) 26:e923015. 10.12659/MSM.923015 32555123 PMC7325558

[36] Tikkanen E Gustafsson S Ingelsson E. Associations of fitness, physical activity, strength, and genetic risk with cardiovascular disease: longitudinal analyses in the uk biobank study. Circulation. (2018) 137:2583–91. 10.1161/CIRCULATIONAHA.117.032432 29632216 PMC5997501

[37] Kandola AA Osborn D Stubbs B Choi KW Hayes JF. Individual and combined associations between cardiorespiratory fitness and grip strength with common mental disorders: a prospective cohort study in the uk biobank. Bmc Med. (2020) 18:303. 10.1186/s12916-020-01782-9 33172457 PMC7656705

[38] Clauss D Tjaden C Hackert T Schneider L Ulrich CM Wiskemann J. Cardiorespiratory fitness and muscle strength in pancreatic cancer patients. Support Care Cancer. (2017) 25:2797–807. 10.1007/s00520-017-3694-8 28417202

[39] Brott T Adams HJ Olinger CP Marler JR Barsan WG Biller J. Measurements of acute cerebral infarction: a clinical examination scale. Stroke. (1989) 20:864–70. 10.1161/01.STR.20.7.864 2749846

[40] Sullivan KJ Tilson JK Cen SY Rose DK Hershberg J Correa A. Fugl-meyer assessment of sensorimotor function after stroke: standardized training procedure for clinical practice and clinical trials. Stroke. (2011) 42:427–32. 10.1161/STROKEAHA.110.592766 21164120

[41] Oldfield RC. The assessment and analysis of handedness: the edinburgh inventory. Neuropsychologia. (1971) 9:97–113. 10.1016/0028-3932(71)90067-4 5146491

[42] van Swieten JC Koudstaal PJ Visser MC Schouten HJ van Gijn J. Interobserver agreement for the assessment of handicap in stroke patients. Stroke. (1988) 19:604–7. 10.1161/01.STR.19.5.604 3363593

[43] Nasreddine ZS Phillips NA Bédirian V Charbonneau S Whitehead V Collin I. The montreal cognitive assessment, moca: a brief screening tool for mild cognitive impairment. J Am Geriatr Soc. (2005) 53:695–9. 10.1111/j.1532-5415.2005.53221.x 15817019

[44] Marsden DL Dunn A Callister R McElduff P Levi CR Spratt NJ. A home- and community-based physical activity program can improve the cardiorespiratory fitness and walking capacity of stroke survivors. J Stroke Cerebrovasc Dis. (2016) 25:2386–98. 10.1016/j.jstrokecerebrovasdis.2016.06.007 27378733

[45] Vahlberg B Lindmark B Zetterberg L Hellström K Cederholm T. Body composition and physical function after progressive resistance and balance training among older adults after stroke: an exploratory randomized controlled trial. Disabil Rehabil. (2017) 39:1207–14. 10.1080/09638288.2016.1191551 27341068

[46] Cheng DK Nelson M Brooks D Salbach NM. Validation of stroke-specific protocols for the 10-meter walk test and 6-minute walk test conducted using 15-meter and 30-meter walkways. Top Stroke Rehabil. (2020) 27:251–61. 10.1080/10749357.2019.1691815 31752634

[47] Celis-Morales CA Lyall DM Anderson J Iliodromiti S Fan Y Ntuk UE. The association between physical activity and risk of mortality is modulated by grip strength and cardiorespiratory fitness: evidence from 498 135 uk-biobank participants. Eur Heart J. (2017) 38:116–22. 10.1093/eurheartj/ehw249 28158566 PMC5837781

[48] Merki-Künzli C Kerstan-Huber M Switalla D Gisi D Raptis DA Greco N. Assessing the value of prehabilitation in patients undergoing colorectal surgery according to the enhanced recovery after surgery (eras) pathway for the improvement of postoperative outcomes: protocol for a randomized controlled trial. JMIR Res Protoc. (2017) 6:e199. 10.2196/resprot.7972 29079551 PMC5681719

[49] Srisim K Saengsuwan J Amatachaya S. Functional assessments for predicting a risk of multiple falls in independent ambulatory patients with spinal cord injury. J Spinal Cord Med. (2015) 38:439–45. 10.1179/2045772313Y.0000000186 24621036 PMC4612199

[50] Yang SH Chung EJ Lee J Lee SH Lee BH. The effect of trunk stability training based on visual feedback on trunk stability, balance, and upper limb function in stroke patients: a randomized control trial. Healthcare. (2021) 9:532. 10.3390/healthcare9050532 34063280 PMC8147414

[51] Duncan PW Weiner DK Chandler J Studenski S. Functional reach: a new clinical measure of balance. J Gerontol. (1990) 45:M192–7. 10.1093/geronj/45.6.M192 2229941

[52] Berg K Wood-Dauphinee S Williams JI. The balance scale: reliability assessment with elderly residents and patients with an acute stroke. Scand J Rehabil Med. (1995) 27:27–36. 7792547

[53] Blum L Korner-Bitensky N. Usefulness of the berg balance scale in stroke rehabilitation: a systematic review. Phys Ther. (2008) 88:559–66. 10.2522/ptj.20070205 18292215

[54] Chan CY Subramaniam S Chin KY Ima-Nirwana S Muhammad N Fairus A. Levels of knowledge, beliefs, and practices regarding osteoporosis and the associations with bone mineral density among populations more than 40 years old in malaysia. Int J Environ Res Public Health. (2019) 16:4115. 10.3390/ijerph16214115 31731507 PMC6861980

[55] Wang YC Bohannon RW Li X Sindhu B Kapellusch J. Hand-grip strength: normative reference values and equations for individuals 18 to 85 years of age residing in the united states. J Orthop Sports Phys Ther. (2018) 48:685–93. 10.2519/jospt.2018.7851 29792107

[56] Marques A Gaspar DMM Henriques-Neto D Peralta M Gouveia ÉR Tesler R. Grip strength and depression symptoms among middle-age and older adults. Mayo Clin Proc. (2020) 95:2134–43. 10.1016/j.mayocp.2020.02.035 33012344

[57] Rikli RE Jones CJ. Development and validation of criterion-referenced clinically relevant fitness standards for maintaining physical independence in later years. Gerontologist. (2013) 53:255–67. 10.1093/geront/gns071 22613940

[58] Steffen TM Hacker TA Mollinger L. Age- and gender-related test performance in community-dwelling elderly people: six-minute walk test, berg balance scale, timed up & go test, and gait speeds. Phys Ther. (2002) 82:128–37. 10.1093/ptj/82.2.128 11856064

[59] Patterson SL Forrester LW Rodgers MM Ryan AS Ivey FM Sorkin JD. Determinants of walking function after stroke: differences by deficit severity. Arch Phys Med Rehabil. (2007) 88:115–9. 10.1016/j.apmr.2006.10.025 17207686

[60] Boss HM Van Schaik SM Witkamp TD Geerlings MI Weinstein HC Van den Berg-Vos RM. Cardiorespiratory fitness, cognition and brain structure after tia or minor ischemic stroke. Int J Stroke. (2017) 12:724–31. 10.1177/1747493017702666 28382852

[61] Thilarajah S Mentiplay BF Bower KJ Tan D Pua YH Williams G. Factors associated with post-stroke physical activity: a systematic review and meta-analysis. Arch Phys Med Rehabil. (2018) 99:1876–89. 10.1016/j.apmr.2017.09.117 29056502

[62] Blokhin IO Lentz SR. Mechanisms of thrombosis in obesity. Curr Opin Hematol. (2013) 20:437–44. 10.1097/MOH.0b013e3283634443 23817170 PMC4445633

[63] Janssen I Katzmarzyk PT Ross R. Waist circumference and not body mass index explains obesity-related health risk. Am J Clin Nutr. (2004) 79:379–84. 10.1093/ajcn/79.3.379 14985210

[64] Li M Wang J Liu F Chen H Lu F Wu G. Handedness- and brain size-related efficiency differences in small-world brain networks: a resting-state functional magnetic resonance imaging study. Brain Connect. (2015) 5:259–65. 10.1089/brain.2014.0291 25535788 PMC4432881

[65] Wang D Buckner RL Liu H. Cerebellar asymmetry and its relation to cerebral asymmetry estimated by intrinsic functional connectivity. J Neurophysiol. (2013) 109:46–57. 10.1152/jn.00598.2012 23076113 PMC3545158

[66] Pardo V Knuth D McDermott B Powell J Goldberg A. Validity, reliability and minimum detectable change of the maximum step length test in people with stroke. J Neurol Sci. (2013) 325:74–8. 10.1016/j.jns.2012.11.021 23269279

[67] Oyama C Otaka Y Onitsuka K Takagi H Tan E Otaka E. Reliability and validity of the japanese version of the mini-balance evaluation systems test in patients with subacute stroke. Prog Rehabil Med. (2018) 3:20180015. 10.2490/prm.20180015 32789240 PMC7365213

[68] Woollacott MH Shumway-Cook A Nashner LM. Aging and posture control: changes in sensory organization and muscular coordination. Int J Aging Hum Dev. (1986) 23:97–114. 10.2190/VXN3-N3RT-54JB-X16X 3557634

[69] Mierau A Pester B Hülsdünker T Schiecke K Strüder HK Witte H. Cortical correlates of human balance control. Brain Topogr. (2017) 30:434–46. 10.1007/s10548-017-0567-x 28466295 PMC5495870

[70] Lam NW Goh HT Kamaruzzaman SB Chin AV Poi PJ Tan MP. Normative data for hand grip strength and key pinch strength, stratified by age and gender for a multiethnic asian population. Singapore Med J. (2016) 57:578–84. 10.11622/smedj.2015164 26768064 PMC5075959

