# Supplementary material for: Exploring Alternative Measurements of Cardiorespiratory Fitness in Patients With Mild Ischemic Stroke at Acute Phase
Source: Front Neurol. 2022 Feb 9;13:801696. doi: 10.3389/fneur.2022.801696 (PMC8864240; doi:10.3389/fneur.2022.801696)
Supplement: Supplementary file 1 [file Table_1.DOCX]

Supplementary Figure 1


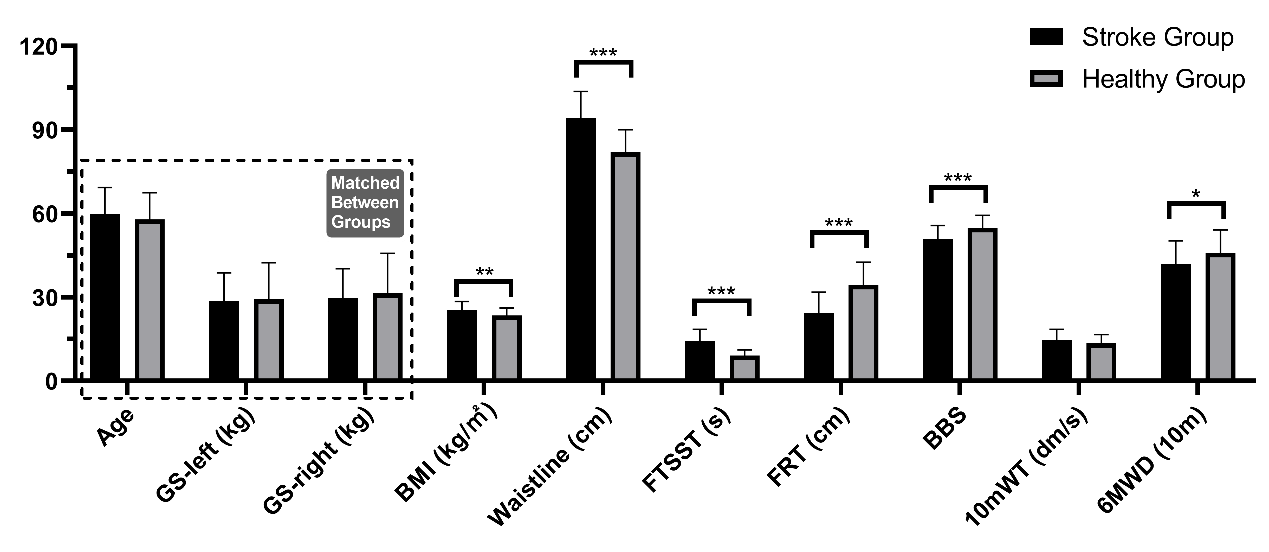


**SUPPLEMENTARY FIGURE 1 |** Comparison of demographic and clinical characteristics related to CRF between the two groups.

BMI, Body Mass Index; GS, grip strength; FTSST, five times sit to stand test; FRT, functional reaching test; BBS, Berg Balance Scale; 10mWT, ten-meter walk test; 6MWD, six-minute walk distance.
